# Depressive Symptoms, sleep Disturbance, and Cardiometabolic Multimorbidity Among Adults With Arthritis: A Cross‐Sectional Analysis of NHANES 2013–2020

**DOI:** 10.1002/kjm2.70284

**Published:** 2026-09-21

**Authors:** Lu‐Niao Ma, Jing‐Cheng Jiang, Jun‐Jie Lan, Yang‐Yang Chen, Hui‐Min Zhang, Yao Zeng, Xu‐Hui Huang, Xiao‐Na Tang

**Affiliations:** ^1^ The Seventh Clinical College of Guangzhou University of Chinese Medicine Shenzhen China; ^2^ Department of Neurosurgery The Second People's Hospital of Yibin Yibin China

**Keywords:** arthritis, CMM, depressive symptoms, sleep disturbance

## Abstract

While the association between depressive symptoms and cardiometabolic disease is well established, the relationships among depressive symptoms, sleep disturbance, and cardiometabolic multimorbidity (CMM) in adults with arthritis remain insufficiently understood. This cross‐sectional study used National Health and Nutrition Examination Survey (NHANES) 2013–2020 data and included 4031 adults with arthritis. Depressive symptoms were assessed using the 9‐item Patient Health Questionnaire (PHQ‐9), and sleep disturbance was defined by self‐reported physician‐diagnosed sleep disorder. CMM was defined as the coexistence of at least two cardiometabolic conditions, including hypertension, diabetes, heart disease, and stroke. Survey‐weighted multivariable logistic regression examined the associations of depressive symptoms and sleep disturbance with CMM. Subgroup analyses and interaction tests evaluated consistency, and an exploratory mediation analysis assessed the role of sleep disturbance in the observed association between depressive symptoms and CMM. In the fully adjusted model, depressive symptoms were associated with higher odds of CMM (odds ratio [OR] = 1.76, 95% confidence interval [CI]: 1.26–2.45, *p* = 0.002), and sleep disturbance was independently associated with CMM (OR = 1.52, 95% CI: 1.24–1.88, *p* < 0.001). The associations remained generally consistent across subgroups. The exploratory mediation analysis suggested that sleep disturbance accounted for approximately 15%–19% of this association. These findings indicate that depressive symptoms and sleep disturbance are independently associated with CMM among adults with arthritis. Sleep disturbance may represent an important co‐occurring factor and warrants further investigation in prospective studies.

## 1 Introduction

Arthritis encompasses a group of acute and chronic joint disorders characterized by pain, swelling, stiffness, joint deformity, and functional limitation [1, 2]. Among the various subtypes, osteoarthritis (OA) and rheumatoid arthritis (RA) are the most common [3, 4]. Arthritis substantially impairs quality of life and is a leading cause of disability among adults in the United States [3, 5]. With global population aging, the prevalence of arthritis is expected to increase further, imposing a considerable socioeconomic burden [6, 7, 8]. Importantly, individuals with arthritis have a substantially higher risk of cardiometabolic diseases and related mortality than age‐ and sex‐matched controls [9, 10, 11]. Chronic systemic inflammation is considered a key mechanism underlying this association, contributing to endothelial dysfunction and accelerated atherosclerosis [12]. In addition, emerging evidence suggests that psychological comorbidities, particularly depression, may further increase cardiovascular risk through complex bidirectional neuroimmune interactions [13, 14].

Cardiometabolic multimorbidity (CMM), commonly defined as the coexistence of two or more cardiometabolic conditions, provides a more comprehensive measure of cardiometabolic disease burden than any single condition alone. This outcome is especially relevant in individuals with arthritis, who frequently experience chronic inflammation, persistent pain, reduced physical activity, and functional impairment, all of which contribute to the clustering of cardiovascular and metabolic disorders [15, 16]. Therefore, examining CMM rather than individual cardiometabolic diseases may better capture the overall cardiometabolic burden in this population and provide a clinically meaningful framework for investigating its associations with depressive symptoms and sleep disturbance.

Depression is a common comorbidity in patients with arthritis and is particularly prevalent among individuals with RA. A meta‐analysis estimated the pooled prevalence of major depressive disorder (MDD) in patients with RA to be approximately 17% [17]. Longitudinal evidence from a Canadian cohort further demonstrated a significantly higher incidence of depression in individuals with RA than in the general population [18]. Clinically, depressive symptoms reduce the likelihood of achieving disease remission and may weaken treatment response [17]. They are also associated with greater pain severity, increased disability, poorer medication adherence, and reduced quality of life [19]. Beyond these clinical consequences, accumulating evidence indicates that depressive symptoms are closely linked to cardiometabolic health. Depression has been associated with an increased risk of myocardial infarction and poorer outcomes following cardiovascular events [20]. Large prospective cohort studies have also identified depression as an independent predictor of incident type 2 diabetes [21]. These findings suggest that depression may contribute to cardiometabolic risk through multiple biological and behavioral pathways and may be particularly important in individuals with arthritis.

Sleep disturbance frequently co‐occurs with depressive symptoms and shares a complex bidirectional relationship with depression [22]. Individuals with depression commonly experience sleep‐related problems, including insomnia, excessive daytime sleepiness, and circadian rhythm disturbances [23]. Conversely, sleep disturbance may increase the risk of developing or worsening depressive symptoms by disrupting emotional regulation, cognitive function, and neuroendocrine balance. Among adults with arthritis, abnormal sleep patterns, including both short and long sleep duration as well as insomnia symptoms, are highly prevalent. Importantly, these sleep disturbances have been independently associated with adverse cardiovascular and metabolic outcomes [24, 25, 26].

Although depression is a well‐established cardiovascular risk factor in the general population, its relationship with cardiometabolic burden among adults with arthritis has not been fully clarified. Unlike the often chronic and progressive course of arthritis, depressive symptoms represent a potentially modifiable risk factor that may be amenable to targeted intervention. Given the potential association between depressive symptoms and CMM, early identification and management of depression may have important clinical implications in this population. However, the role of sleep disturbance in this relationship remains unclear, and evidence among adults with arthritis is limited. Therefore, using data from the National Health and Nutrition Examination Survey (NHANES), we examined the association between depressive symptoms and CMM among adults with arthritis. We also evaluated the association between sleep disturbance and CMM and conducted an exploratory mediation analysis to assess the potential role of sleep disturbance in the observed association between depressive symptoms and CMM.

## 2 Methods

### 2.1 Study Design and Participants

This cross‐sectional study used data from the NHANES, an ongoing program that has monitored the health and nutritional status of the US population since 1999 [23]. NHANES employs a stratified, multistage probability sampling design and, when appropriate sampling weights and survey design variables are applied, provides nationally representative estimates of the civilian, noninstitutionalized US population. The survey protocol was approved by the National Center for Health Statistics Research Ethics Review Board, and written informed consent was obtained from all participants. Because NHANES data are publicly available and de‐identified, this secondary analysis was exempt from additional institutional review board approval. All analyses were conducted in accordance with NHANES analytic guidelines. Data from four NHANES cycles (2013–March 2020) were included because these cycles contained the key variables required for the present study, including measures of sleep disturbance, the 9‐item Patient Health Questionnaire (PHQ‐9) scores, and components used to define CMM.

**FIGURE 1:**
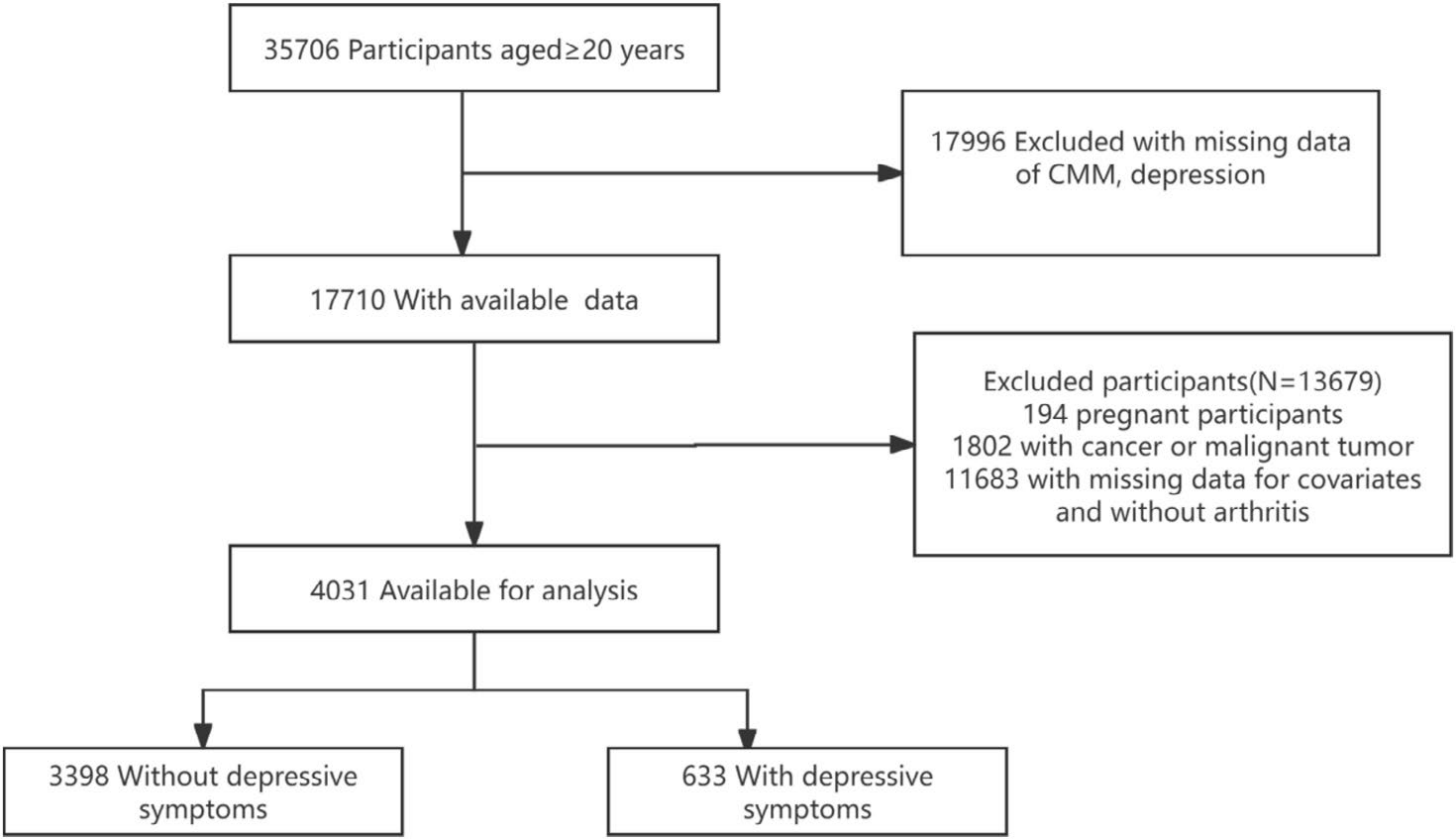
Flowchart illustrating the selection of study participants. CMM, cardiometabolic multimorbidity.

The participant selection process is illustrated in Figure 1. A total of 35,706 individuals aged ≥ 20 years were initially identified from the NHANES database. Participants were subsequently excluded if they had missing PHQ‐9 or CMM data (*n* = 17,996), a history of malignancy (*n* = 1802), were pregnant (*n* = 194), or did not have arthritis or had incomplete covariate data (*n* = 11,683). After applying these exclusion criteria, 4031 adults with arthritis were included in the final analytic sample.

### 2.2 Definition of CMM

CMM was defined as the coexistence of at least two of the following four chronic conditions: hypertension, diabetes, heart disease, and stroke. The presence of each condition was determined based on participants' self‐reported physician diagnoses recorded in NHANES. Hypertension was identified by an affirmative response to the question, “Has a doctor ever told you that you have hypertension?” Diabetes was defined by a positive response to the question, “Have you ever been diagnosed with diabetes or elevated blood glucose, including impaired glucose tolerance or impaired fasting glucose?” Heart disease was defined as a self‐reported physician diagnosis of myocardial infarction, coronary heart disease, angina, congestive heart failure, or another heart condition. Stroke was identified by an affirmative response to the question, “Have you ever been diagnosed by a doctor with a stroke, including ischemic stroke or intracerebral hemorrhage?”

### 2.3 Definition of Depressive Symptoms and Sleep Disturbance

Depressive symptoms were assessed using the PHQ‐9, a validated screening instrument widely used to evaluate depressive symptoms in the general population [27]. The PHQ‐9 comprises nine items, each scored on a 4‐point scale ranging from 0 (“not at all”) to 3 (“nearly every day”), resulting in a total score ranging from 0 to 27. Participants with a PHQ‐9 score ≥ 10 were classified as having clinically significant depressive symptoms. Sleep disturbance was assessed using the NHANES sleep questionnaire. Participants who reported that a doctor or other healthcare professional had informed them that they had a sleep disorder were classified as having sleep disturbance.

### 2.4 Definition of Arthritis

Arthritis was identified using the NHANES Medical Conditions Questionnaire. Participants who responded “yes” to the question asking whether a doctor or other healthcare professional had ever told them that they had arthritis were classified as having arthritis. NHANES further collected self‐reported information on arthritis subtype, including OA or degenerative arthritis, RA, psoriatic arthritis, and other forms of arthritis. However, because arthritis subtype was self‐reported, the distribution of subtypes was uneven, and a substantial proportion of participants reported that they did not know their arthritis type. Therefore, adults with arthritis were analyzed as a single group rather than stratified by specific arthritis subtypes.

### 2.5 Definition of Covariates

Covariates were selected a priori based on previous literature and clinical relevance, with particular consideration of factors associated with depressive symptoms or cardiometabolic risk. The covariates included sociodemographic characteristics (age, sex, race/ethnicity, educational attainment, marital status, and poverty income ratio [PIR]), lifestyle factors (smoking status, alcohol consumption, and physical activity), and clinical or laboratory variables (body mass index [BMI], total cholesterol, platelet count, lymphocyte count, and white blood cell count).

Sex was categorized as male or female. Educational attainment was classified as below high school, high school, or above high school. Race/ethnicity was categorized as Mexican American, non‐Hispanic White, non‐Hispanic Black, or other race/ethnicity. Marital status was classified as married or living with a partner, single, divorced, or widowed. PIR was categorized as low (< 1.30), medium (1.31–3.50), or high (> 3.50). Smoking status was determined according to self‐reported lifetime cigarette consumption of at least 100 cigarettes and current smoking status. Participants who reported consuming at least 12 alcoholic drinks in any 1‐year period were classified as alcohol drinkers [28]. Physical activity was categorized as sedentary, moderate, or vigorous.

### 2.6 Statistical Analysis

Categorical variables are presented as frequencies and survey‐weighted percentages, whereas continuous variables are reported as survey‐weighted means with standard errors (SEs). Between‐group comparisons were performed using Rao–Scott chi‐square tests for categorical variables and design‐based Wald tests for continuous variables.

Survey‐weighted multivariable logistic regression analyses were conducted to examine the associations of depressive symptoms and sleep disturbance with CMM. Three hierarchical models were constructed to account for potential confounding factors: Model 1 was unadjusted; Model 2 was adjusted for age, sex, and race/ethnicity; and Model 3 was additionally adjusted for educational attainment, marital status, PIR, smoking status, alcohol consumption, BMI, physical activity, platelet count, lymphocyte count, white blood cell count, and total cholesterol. The results are presented as ORs with 95% confidence intervals (CIs).

An exploratory mediation analysis was subsequently performed to evaluate the potential role of sleep disturbance in the observed association between depressive symptoms and CMM. Bootstrap resampling with 5000 iterations was used to estimate 95% CIs, applying the same covariate‐adjustment strategy used in the multivariable regression models. In addition, survey‐weighted stratified analyses were conducted across prespecified subgroups defined by sex, age (< 65 vs. ≥ 65 years), PIR, BMI, physical activity level, smoking status, and alcohol consumption to assess the consistency of the observed associations.

All statistical analyses were performed using R software (version 4.2.2; R Foundation for Statistical Computing, Vienna, Austria) and Free Statistics software (version 2.1.1). The complex sampling design of NHANES was accounted for in R using the “survey” package, incorporating sampling weights, strata, and primary sampling units. A two‐sided *p* < 0.05 was considered statistically significant.

## 3 Results

### 3.1 Baseline Characteristics

A total of 4031 adults with arthritis were included in the analysis, of whom 633 had depressive symptoms and 3398 did not. The baseline characteristics of the study population are presented in Table 1. Compared with participants without depressive symptoms, those with depressive symptoms were significantly younger, more likely to be female, and more frequently single, divorced, or widowed. They also had lower educational attainment and lower PIR, whereas race/ethnicity distributions were comparable between the two groups. With respect to lifestyle factors, sleep disturbance was substantially more prevalent among participants with depressive symptoms than among those without depressive symptoms (77.6% vs. 46.1%, *p* < 0.001). Participants with depressive symptoms were also more likely to be current smokers and alcohol drinkers and exhibited significantly different patterns of physical activity (*p* < 0.001 for all). Clinically, they had a higher BMI and a greater prevalence of CMM than their counterparts without depressive symptoms. In addition, platelet count, lymphocyte count, and white blood cell count were modestly but significantly higher among participants with depressive symptoms (*p* < 0.05 for all).

**TABLE 1:** Weighted characteristics of participants with and without depressive symptoms.

Variables	Total (*n* = 4031)	Depressive symptoms		*p*
		No (*n* = 3398)	Yes (*n* = 633)	
Sex, *n* (weighted %)				**0.003**
Male	1632 (39.5)	1416 (40.9)	216 (30.9)	
Female	2399 (60.5)	1982 (59.1)	417 (69.1)	
Age, years, weighted mean (SE)	58.47 (0.31)	58.84 (0.31)	56.30 (0.79)	0.003
Race, *n* (weighted %)				**0.116**
Non‐Hispanic White	1736 (71.5)	1472 (72.2)	264 (67.3)	
Non‐Hispanic Black	1056 (11.4)	897 (11.2)	159 (12.7)	
Mexican American	437 (5.0)	356 (4.9)	81 (5.8)	
Other race/ethnicity	802 (12.2)	673 (11.8)	129 (14.2)	
Marital status, *n* (weighted %)				**0.016**
Married or living with a partner	2266 (62.5)	1943 (63.5)	323 (56.0)	
Single, divorced, or widowed	1765 (37.5)	1455 (36.5)	310 (44.0)	
Education level, *n* (weighted %)				**< 0.001**
< 9 years	392 (4.5)	297 (3.9)	95 (8.0)	
9–12 years	1546 (36.4)	1265 (34.9)	281 (45.2)	
> 12 years	2093 (59.1)	1836 (61.2)	257 (46.8)	
PIR, *n* (weighted %)				**< 0.001**
≤ 1.30	1366 (22.6)	1041 (19.7)	325 (40.2)	
1.31–3.50	1527 (36.2)	1303 (35.7)	224 (38.6)	
> 3.50	1138 (41.2)	1054 (44.6)	84 (21.2)	
Sleep disturbance, *n* (weighted %)				**< 0.001**
No	2114 (49.4)	1955 (53.9)	159 (22.4)	
Yes	1917 (50.6)	1443 (46.1)	474 (77.6)	
Physical activity, *n* (weighted %)				**< 0.001**
Sedentary	2470 (55.2)	1988 (52.9)	482 (69.2)	
Moderate	1029 (28.8)	912 (29.8)	117 (22.7)	
Vigorous	532 (16.0)	498 (17.3)	34 (8.1)	
Smoking status, *n* (weighted %)				**< 0.001**
Never	1940 (47.7)	1688 (49.5)	252 (37.2)	
Former	1246 (32.4)	1060 (32.9)	186 (29.6)	
Current	845 (19.9)	650 (17.7)	195 (33.2)	
Alcohol consumption, *n* (weighted %)				**< 0.001**
No	3228 (81.1)	2785 (83.0)	443 (69.5)	
Yes	803 (18.9)	613 (17.0)	190 (30.5)	
BMI, kg/m^2^, weighted mean (SE)	31.44 (0.18)	31.12 (0.20)	33.32 (0.51)	< 0.001
CMM, *n* (weighted %)				**< 0.001**
No	1498 (43.2)	1333 (45.6)	165 (28.8)	
Yes	2533 (56.8)	2065 (54.4)	468 (71.2)	
Platelet count, ×10^9^/L, weighted mean (SE)	239.99 (1.33)	238.76 (1.39)	247.27 (3.76)	0.038
Lymphocyte count, ×10^9^/L, weighted mean (SE)	2.11 (0.02)	2.08 (0.02)	2.25 (0.06)	0.007
White blood cell count, ×10^9^/L, weighted mean (SE)	7.39 (0.06)	7.33 (0.07)	7.75 (0.15)	0.012
Total cholesterol, mmol/L, weighted mean (SE)	4.97 (0.03)	4.97 (0.04)	5.00 (0.09)	0.794

*Note:* Bold values indicate *p* < 0.05.

Abbreviations: BMI, body mass index; CMM, cardiometabolic multimorbidity; PIR, poverty income ratio; SE, standard error.

### 3.2 Associations of Sleep Disturbance and Depressive Symptoms With CMM

The associations of sleep disturbance and depressive symptoms with CMM are presented in Table 2. In the unadjusted model (Model 1), both sleep disturbance and depressive symptoms were significantly associated with increased odds of CMM. These associations remained significant after adjustment for potential confounders. In the fully adjusted model (Model 3), participants with sleep disturbance had 52% higher odds of CMM than those without sleep disturbance (OR = 1.52, 95% CI: 1.24–1.88, *p* < 0.001). Similarly, depressive symptoms were independently associated with higher odds of CMM (OR = 1.76, 95% CI: 1.26–2.45, *p* = 0.002).

**TABLE 2:** Survey‐weighted associations of depressive symptoms and sleep disturbance with cardiometabolic multimorbidity.

Exposure	Model 1 OR (95% CI)	SE of *β*	*p*	Model 2 OR (95% CI)	SE of *β*	*p*	Model 3 OR (95% CI)	SE of *β*	*p*
Depressive symptoms	1.86 (1.38–2.50)	0.148	< 0.001	2.18 (1.58–3.00)	0.160	< 0.001	1.76 (1.26–2.45)	0.164	0.002
Sleep disturbance	1.43 (1.18–1.73)	0.095	< 0.001	1.60 (1.30–1.97)	0.104	< 0.001	1.52 (1.24–1.88)	0.102	< 0.001

*Note:* Odds ratios, 95% confidence intervals, standard errors, and *p* values were estimated using survey‐weighted logistic regression models accounting for the NHANES sampling weights, strata, and primary sampling units. SE denotes the design‐based standard error of the log‐odds coefficient (*β*).

Model 1 included depressive symptoms and sleep disturbance simultaneously without additional covariates. Model 2 was additionally adjusted for age, sex, and race/ethnicity. Model 3 was further adjusted for marital status, education level, poverty income ratio, physical activity, smoking status, alcohol consumption, body mass index, platelet count, lymphocyte count, white blood cell count, and total cholesterol.

We further examined the survey‐weighted joint association of sleep disturbance and depressive symptoms with CMM in Table 3. Compared with participants with neither depressive symptoms nor sleep disturbance, those with sleep disturbance only had significantly higher odds of CMM (OR = 1.51, 95% CI: 1.23–1.86, *p* < 0.001). The association for depressive symptoms only did not reach statistical significance in the fully adjusted model (OR = 1.64, 95% CI: 0.98–2.73, *p* = 0.058), whereas participants with both depressive symptoms and sleep disturbance had the highest odds of CMM (OR = 2.72, 95% CI: 1.80–4.12, *p* < 0.001). No significant multiplicative interaction was observed between the two factors (OR = 1.10, 95% CI: 0.63–1.92, *p* for interaction = 0.725). On the additive scale, the RERI and AP estimates were greater than 0 and the SI was greater than 1, indicating concordance in the direction of the three interaction measures. The RERI was 0.02 (95% CI: −0.18 to 0.22), the AP was 0.02 (95% CI: −0.13 to 0.16), and the SI was 1.06 (95% CI: 0.61–1.84). However, all estimates were close to their corresponding null values, and all 95% CIs included the null values. Therefore, no statistically significant evidence of additive interaction was observed. The underlying survey‐weighted standardized prevalence estimates and prevalence ratios for the four joint exposure categories are provided in Table S1.

**TABLE 3:** Survey‐weighted joint and interactive associations of depressive symptoms and sleep disturbance with cardiometabolic multimorbidity.

Panel A. Joint exposure analysis									
Joint exposure category	Model 1			Model 2			Model 3		
	OR (95% CI)	SE of *β*	*p*	OR (95% CI)	SE of *β*	*p*	OR (95% CI)	SE of *β*	*p*
Neither depressive symptoms nor sleep disturbance	1.00 (Reference)	—	—	1.00 (Reference)	—	—	1.00 (Reference)	—	—
Sleep disturbance only	1.44 (1.19–1.75)	0.097	< 0.001	1.60 (1.29–1.97)	0.106	< 0.001	1.51 (1.23–1.86)	0.102	< 0.001
Depressive symptoms only	2.02 (1.27–3.22)	0.232	0.004	2.11 (1.26–3.52)	0.255	0.005	1.64 (0.98–2.73)	0.251	0.058
Both depressive symptoms and sleep disturbance	2.60 (1.83–3.70)	0.176	< 0.001	3.52 (2.42–5.10)	0.185	< 0.001	2.72 (1.80–4.12)	0.203	< 0.001

Panel B. Multiplicative interaction				
Interaction term	Fully adjusted survey‐weighted interaction model	OR (95% CI)	SE of *β*	*p* for interaction
Depressive symptoms × sleep disturbance		1.10 (0.63–1.92)	0.273	0.725

Panel C. Additive interaction		
Additive interaction measure	Estimate	95% CI
RERI based on standardized prevalence ratios	0.02	−0.18 to 0.22
AP due to interaction	0.02	−0.13 to 0.16
SI	1.06	0.61 to 1.84

*Note:* Model 1 was unadjusted. Model 2 was adjusted for age, sex, and race/ethnicity. Model 3 was additionally adjusted for marital status, education level, poverty income ratio, physical activity, smoking status, alcohol consumption, body mass index, platelet count, lymphocyte count, white blood cell count, and total cholesterol. SE of *β* denotes the design‐based standard error of the log‐odds coefficient. In Panel C, RERI, AP, and SI with their 95% CIs were derived from survey‐weighted standardized prevalences obtained using predictive margins from Model 3 and the survey‐design covariance matrix. Null values are 0 for RERI and AP and 1 for SI.

Abbreviations: AP, attributable proportion due to interaction; CI, confidence interval; CMM, cardiometabolic multimorbidity; OR, odds ratio; RERI, relative excess risk due to interaction; SE, standard error; SI, synergy index.

### 3.3 Subgroup Analysis

**FIGURE 2:**
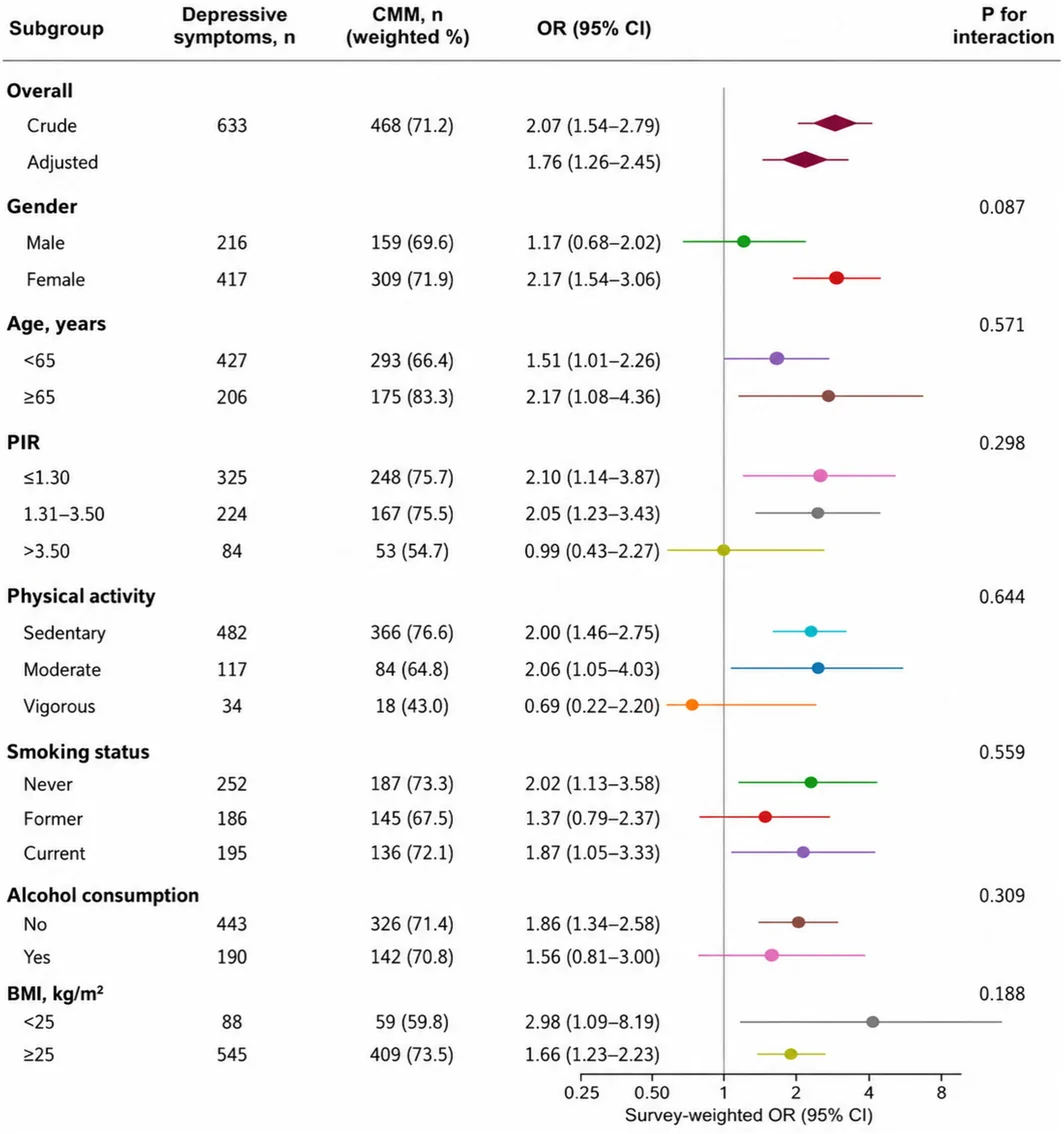
Survey‐weighted subgroup analyses of the association between depressive symptoms and cardiometabolic multimorbidity. The crude model was unadjusted. The adjusted model included sleep disturbance, age, sex, race/ethnicity, educational attainment, marital status, poverty income ratio, smoking status, alcohol consumption, body mass index, physical activity, platelet count, lymphocyte count, white blood cell count, and total cholesterol. Subgroup‐specific models were adjusted for all covariates except the variable used to define the corresponding subgroup. BMI, body mass index; CMM, cardiometabolic multimorbidity; CI, confidence interval; OR, odds ratio; PIR, poverty income ratio.

Survey‐weighted stratified analyses were performed to assess whether the association between depressive symptoms and CMM was consistent across prespecified subgroups. As shown in Figure 2, depressive symptoms were associated with significantly higher odds of CMM in the fully adjusted model (overall OR = 1.76, 95% CI: 1.26–2.45). This positive association was generally consistent across subgroups defined by sex, age, PIR, physical activity level, smoking status, alcohol consumption, and BMI, with all *p* values for interaction exceeding 0.05. Although the association did not reach statistical significance in certain subgroups, including men, participants with a PIR > 3.50, those engaging in vigorous physical activity, former smokers, and alcohol drinkers, most subgroup‐specific estimates were in the same positive direction as the overall association. However, the estimates for participants with a PIR > 3.50 and those engaging in vigorous physical activity were close to or below the null and had wide CIs. Overall, given that all *p* values for interaction were greater than 0.05, there was no statistical evidence that the association between depressive symptoms and CMM differed across the examined subgroups.

### 3.4 Exploratory Mediation Analysis

**FIGURE 3:**
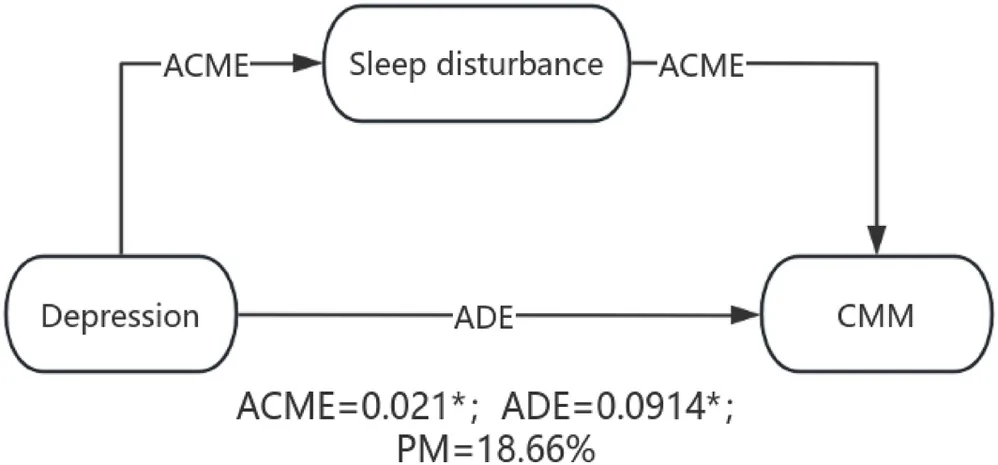
Exploratory mediation analysis of the associations among depressive symptoms, sleep disturbance, and CMM. This model was used to evaluate the potential role of sleep disturbance in the observed association between depressive symptoms and CMM. Given the cross‐sectional design of the study, the estimates should be interpreted as exploratory statistical associations rather than evidence of causal or temporal relationships. ACME, average causal mediation effect; ADE, average direct effect; CMM, cardiometabolic multimorbidity; PM, proportion mediated.

An exploratory mediation analysis was performed to assess the potential role of sleep disturbance in the observed association between depressive symptoms and CMM. In the fully adjusted model, the ACME was 0.0210 (95% CI: 0.0125–0.0322; *p* < 0.001), indicating that sleep disturbance was associated with a small but statistically significant proportion of the observed association between depressive symptoms and CMM. The average direct effect (ADE) remained statistically significant after accounting for sleep disturbance (ADE = 0.0914, 95% CI: 0.0480–0.1327; *p* < 0.001). Likewise, the total effect estimates were significant in all three models (Model 1: 0.1414; Model 2: 0.1648; Model 3: 0.1123; all *p* < 0.001). The estimated proportion associated with sleep disturbance ranged from 14.5% to 18.7% across the models. Results from the fully adjusted model are illustrated in Figure 3. Overall, the exploratory mediation analysis suggested that sleep disturbance was associated with a modest proportion of the observed association between depressive symptoms and CMM. However, given the cross‐sectional design of the study, these findings should be interpreted as exploratory statistical associations rather than evidence of a causal or temporal pathway.

## 4 Discussion

The present study used a large sample of adults with arthritis from NHANES to investigate the associations among depressive symptoms, sleep disturbance, and CMM, while accounting for the complex survey design of NHANES. First, both depressive symptoms and sleep disturbance were significantly associated with higher odds of CMM. These associations remained significant after adjustment for potential confounders and were generally consistent across the prespecified subgroups. Second, no significant multiplicative interaction was observed between depressive symptoms and sleep disturbance. On the additive scale, all three interaction measures were directionally concordant but close to their respective null values, and none reached statistical significance, providing no evidence of a statistically significant additive interaction. Third, the exploratory mediation analysis indicated that sleep disturbance was associated with a modest proportion of the observed association between depressive symptoms and CMM, accounting for approximately 14.5%–18.7% of the total observed association. Given the cross‐sectional nature of the study, these findings should be interpreted as exploratory associations rather than evidence of a causal or temporal pathway.

The increased cardiometabolic burden observed among participants with depressive symptoms may reflect several interrelated mechanisms, including chronic inflammation, behavioral changes associated with depression, and the underlying inflammatory and functional consequences of arthritis. This interpretation is supported by the higher white blood cell, lymphocyte, and platelet counts observed among participants with depressive symptoms in the present study [19, 29]. Persistent low‐grade inflammation has been implicated in endothelial dysfunction and metabolic dysregulation, both of which contribute to the development of cardiometabolic disease [11]. In addition to inflammatory pathways, lifestyle‐related factors may further increase cardiometabolic risk [30, 31]. Depressive symptoms, together with arthritis‐related pain and functional impairment, may discourage physical activity and promote unhealthy behaviors. Consistent with this hypothesis, participants with depressive symptoms in our study were more likely to report sedentary lifestyles, smoking, alcohol consumption, and higher BMI values. In adults with arthritis, who already face limitations in physical functioning, these adverse lifestyle factors may further compound cardiometabolic vulnerability [32].

Another important aspect of this study is the exploratory evaluation of the potential role of sleep disturbance in the observed association between depressive symptoms and CMM among adults with arthritis. The exploratory mediation analysis indicated that sleep disturbance was associated with approximately 15%–19% of the observed association. This finding is biologically plausible. Depressive symptoms have been linked to dysregulation of the hypothalamic–pituitary–adrenal axis and alterations in cortisol secretion patterns, both of which may contribute to sleep disturbance [33, 34]. In turn, sleep disturbance has been associated with sympathetic nervous system activation, elevated levels of proinflammatory cytokines, insulin resistance, and an increased risk of cardiometabolic conditions such as hypertension and diabetes [35]. Nevertheless, the association between depressive symptoms and CMM remained significant after accounting for sleep disturbance, indicating that additional mechanisms may contribute to this relationship. Potential pathways include autonomic dysfunction, persistent systemic inflammation, and the metabolic effects associated with certain antidepressant medications [36]. Given the cross‐sectional design of the present study, these findings should be interpreted cautiously and regarded as exploratory associations rather than evidence of a causal or temporal pathway.

Taken together, these findings suggest that focusing solely on articular manifestations may not adequately reflect the overall health burden experienced by adults with arthritis. In addition to managing joint‐related symptoms, rheumatologists and primary care physicians may consider routinely assessing depressive symptoms and sleep disturbance as part of comprehensive patient care. Early identification of these conditions may help recognize individuals with a greater cardiometabolic burden. Integrating mental health support and sleep‐focused interventions into conventional arthritis management may provide a more holistic approach to care and potentially improve overall health outcomes in this population.

This study has several strengths. First, it was based on a large sample of adults with arthritis from NHANES, a population known to have a substantial cardiometabolic burden. By focusing specifically on individuals with arthritis, this study extends previous research that has primarily examined general populations or individual cardiometabolic risk factors and provides additional insight into the interrelationships among depressive symptoms, sleep disturbance, and CMM in this clinically important group. Second, a comprehensive analytical approach was employed, including survey‐weighted multivariable adjustment, subgroup analyses, interaction testing, and exploratory mediation analysis. This approach enabled the evaluation of the adjusted associations of depressive symptoms and sleep disturbance with CMM while also exploring the potential role of sleep disturbance in the observed association between depressive symptoms and CMM, thereby providing a more nuanced interpretation of the findings. Furthermore, depressive symptoms and sleep disturbance are common, clinically recognizable conditions that can be assessed using simple, inexpensive, and widely available screening tools. Routine assessment of these factors in adults with arthritis may facilitate the identification of individuals with a greater cardiometabolic burden and support more comprehensive clinical evaluation and management.

Several limitations should also be acknowledged. First, because of the cross‐sectional design, causal relationships cannot be inferred, and the temporal sequence among depressive symptoms, sleep disturbance, and CMM cannot be established. Accordingly, the exploratory mediation findings should be interpreted as statistical associations rather than evidence of a causal mediation mechanism. Longitudinal studies are needed to clarify the directionality and temporal relationships of these associations. Second, depressive symptoms and sleep disturbance were assessed using self‐reported measures, which may be subject to recall and reporting bias, potentially resulting in misclassification and influencing the observed associations. Third, arthritis status was determined based on self‐reported physician diagnoses, and all arthritis subtypes were analyzed as a single group. This approach may have obscured clinically important heterogeneity across arthritis subtypes. RA is characterized by systemic inflammation and may be associated with a cardiometabolic risk profile distinct from that of OA, which is predominantly degenerative and substantially more prevalent. Therefore, the pooled estimates may have been driven largely by participants with OA, potentially diluting associations specific to RA. Although NHANES collected information on arthritis subtype, these data were also self‐reported, and participants may not reliably distinguish RA from OA, which could introduce substantial subtype misclassification. Given the potential unreliability of self‐reported subtype classification, we did not conduct arthritis subtype‐stratified analyses. Future studies using clinically confirmed arthritis subtype diagnoses are warranted to determine whether the observed associations differ across arthritis subtypes. Finally, although the analyses were adjusted for a broad range of sociodemographic, lifestyle, anthropometric, and laboratory covariates, residual confounding due to unmeasured or imperfectly measured factors cannot be excluded.

## 5 Conclusion

In conclusion, among adults with arthritis from NHANES, depressive symptoms and sleep disturbance were significantly associated with higher odds of CMM, whereas no significant multiplicative or additive interaction was observed. The exploratory mediation analysis suggested that sleep disturbance statistically accounted for a modest proportion of the observed association between depressive symptoms and CMM, although causal or temporal interpretations are not warranted. Future prospective and interventional studies are needed to clarify the temporal relationships among depressive symptoms, sleep disturbance, and CMM and to determine whether integrated strategies targeting both mood and sleep can help reduce cardiometabolic burden in this population.

## Funding

This work was supported by Sanming Project of Medicine in Shenzen (No. SZZYSM202206014).

## Ethics Statement

NHANES was approved by the National Health Statistics Research Ethics Review Board. And the collection of this data was approved by an ethical review committee, all participants were provided written informed consent prior to participating in NHANES.

## Conflicts of Interest

The authors declare no conflicts of interest.

## Supporting information


**Table S1:** Fully adjusted survey‐weighted standardized prevalence and prevalence ratios of cardiometabolic multimorbidity according to joint exposure to depressive symptoms and sleep disturbance.

## Data Availability

The data supporting the findings of this study are publicly available from NHANES at https://www.cdc.gov/nchs/nhanes/.
