# Supplementary material for: Depressive Symptoms, sleep Disturbance, and Cardiometabolic Multimorbidity Among Adults With Arthritis: A Cross‐Sectional Analysis of NHANES 2013–2020
Source: Kaohsiung J Med Sci. 2026 Sep 21:e70284. Online ahead of print. doi: 10.1002/kjm2.70284 (PMC13591341; doi:10.1002/kjm2.70284)
Supplement: Supplementary file 1 [file KJM2-9999-e70284-s001.docx]

**Supplementary Table S1. Fully adjusted survey-weighted standardized prevalence and prevalence ratios of cardiometabolic multimorbidity according to joint exposure to depressive symptoms and sleep disturbance**

| **Joint exposure category** | **Unweighted n** | **Adjusted standardized prevalence, % (95% CI)** | **Adjusted standardized prevalence ratio (95% CI)** |
| --- | --- | --- | --- |
| Neither depressive symptoms nor sleep disturbance | 1955 | 50.99 (47.94–54.03) | 1.00 (Reference) |
| Sleep disturbance only | 1443 | 59.43 (55.91–62.95) | 1.17 (1.08–1.26) |
| Depressive symptoms only | 159 | 61.04 (51.55–70.53) | 1.20 (1.02–1.41) |
| Both depressive symptoms and sleep disturbance | 474 | 70.58 (64.11–77.05) | 1.38 (1.24–1.55) |

Counts are unweighted. Adjusted standardized prevalences and prevalence ratios were estimated using predictive margins from a fully adjusted survey-weighted quasibinomial logistic regression model accounting for the cycle-combined MEC examination weights, strata, and primary sampling units. The final arthritis sample was analyzed as a subpopulation of the full survey design. The model was adjusted for age, gender, race/ethnicity, marital status, educational attainment, poverty income ratio, physical activity, smoking status, alcohol consumption, body mass index, platelet count, lymphocyte count, white blood cell count, and total cholesterol. Participants with neither depressive symptoms nor sleep disturbance were used as the reference group. Because this was a cross-sectional analysis, relative estimates are reported as standardized prevalence ratios rather than prospective risk ratios.

*Abbreviations: BMI, body mass index; CI, confidence interval; CMM, cardiometabolic multimorbidity; MEC, Mobile Examination Center; NHANES, National Health and Nutrition Examination Survey; PIR, poverty income ratio; PSU, primary sampling unit.*
